# A Unique SARS-CoV-2 Spike Protein P681H Variant Detected in Israel

**DOI:** 10.3390/vaccines9060616

**Published:** 2021-06-08

**Authors:** Neta S. Zuckerman, Shay Fleishon, Efrat Bucris, Dana Bar-Ilan, Michal Linial, Itay Bar-Or, Victoria Indenbaum, Merav Weil, Yaniv Lustig, Ella Mendelson, Michal Mandelboim, Orna Mor, Neta Zuckerman, on behalf of the Israel National Consortium for SARS-CoV-2 Sequencing

**Affiliations:** 1 Central Virology Laboratory, Israel Ministry of Health, Sheba Medical Center, Tel-Hashomer 52621, Israel; s.fleishon@gmail.com (S.F.); efrat.bucris@sheba.health.gov.il (E.B.); dana.barilan@sheba.health.gov.il (D.B.-I.); itay.baror@sheba.health.gov.il (I.B.-O.); viki.indenbaum@sheba.health.gov.il (V.I.); merav.weil@sheba.health.gov.il (M.W.); yaniv.lustig@sheba.health.gov.il (Y.L.); ella.mendelson@sheba.health.gov.il (E.M.); michal.mandelboim@sheba.health.gov.il (M.M.); orna.mor@sheba.health.gov.il (O.M.); tozaot.negifim@sheba.health.gov.il (N.Z.); 2 Department of Biological Chemistry, The Alexander Silberman Institute of Life Sciences, The Hebrew University of Jerusalem, Jerusalem 91904, Israel; michall@mail.huji.ac.il; 3 Department of Epidemiology and Preventive Medicine, School of Public Health, Sackler Faculty of Medicine, Tel-Aviv University, Tel-Aviv 69978, Israel

**Keywords:** SARS-CoV-2, sequencing, virus neutralization, Covid-19, variant of concern

## Abstract

The routine detection, surveillance, and reporting of novel SARS-CoV-2 variants is crucial, as these threaten to hinder global vaccination efforts. Herein we report a novel local variant with a non-synonymous mutation in the spike (S) protein P681H. This local Israeli variant was not associated with a higher infection rate or higher prevalence. Furthermore, the local variant was successfully neutralized by sera from fully vaccinated individuals at a comparable level to the B.1.1.7 variant and an Israel wild-type strain. While it is not a variant of concern, routine monitoring by sequencing is still required.

## 1. Introduction

SARS-CoV-2 (severe acute respiratory syndrome coronavirus 2) emerged in Wuhan, China in December 2019 [1] and has rapidly spread worldwide, causing severe respiratory disease and leading to substantial morbidity and mortality.

Global whole genome sequencing (WGS) efforts and initiatives, such as the GISAID (global initiative on sharing all influenza data) platform [2], Nextstrain [3], and Pangolin [4,5], facilitate access to worldwide SARS-CoV-2 sequences, tracking different lineages of the virus and detecting emerging novel virus variants. Recently, various variants of concern (VOC) have been identified globally, including the B.1.1.7 variant identified in the UK [6], the B.1.351 variant identified in South Africa [7], and the P.1 variant identified in Brazil [8]. The VOCs are fast spreading and characterized by higher infection rates or lower neutralization in convalescent or vaccinated individuals, consistent with evolutionary advantages for these variants [9,10]. 

Mutations in the spike (S) protein of SARS-CoV-2 attract much attention in monitoring emerging strains, as infection is driven by the S protein binding the cell surface angiotensin-converting enzyme 2 (ACE2) receptor on human cells [11]. Mutations in the SARS-CoV-2 genome affecting the S protein have emerged in numerous differentially-reported variants, such as the N501Y mutation that is shared by the currently known VOCs, B.1.1.7, B.1.351, and P.1, and which increase the affinity of the S protein to its receptor binding domain (RBD) [12]. Similarly, position 681 in the S protein, located in the vicinity of a critical furin cleavage site, has been found to be mutated in several variants. S protein cleavage (after R685 residue) leads to S protein activation by separating the protein to S1 and S2 subunits. Following the RBD binding to the ACE2 receptor, the S1/S2 activation step leads to membrane fusion and the entering of the viral genome into human cells [13]. 

The P681H mutation was observed as early as March 2020 in samples worldwide, such as in Nigeria [3], Hawaii [4], and recently in three independent variants in New York [14], and also characterizes the globally spreading B.1.1.7 VOC [6]. It is postulated that the P681H mutation enhances the transmissibility of the virus by facilitating a conformational change in the S protein following protease activity at the cell membrane [15]. Additionally, the A.23.1 variant that has been reported as the dominant lineage in Uganda [16] and the B.1.617 family of variants identified in India (not published) acquired the P681R mutation in the same position.

In this study, we report a novel variant that emerged from a locally circulating lineage, B.1.1.50. This variant is characterized by the non-synonymous S protein mutation P681H and four additional synonymous mutations. This B.1.1.50 + P681H variant was identified in clinical samples in Israel between November 2020 and January 2021, whereas the P681H mutation had already been identified in Israel via sequencing of SARS-CoV-2 positive sewage samples in October 2020. This novel variant has not been associated with a higher infection rate and its frequency has declined in correlation with the increase of the B.1.1.7 variant in Israel. Neutralization assays with sera from vaccinated individuals showed comparable neutralization levels to the B.1.1.7 variant and an Israel wild-type (WT) strain. 

With increasing reports on emerging VOC worldwide threatening to hinder global vaccination efforts, the routine surveillance and reporting of local variants, which have the potential for spreading globally, is extremely important in the battle against Covid-19 and further spreading of SARS-CoV-2.

## 2. Materials and Methods

### 2.1. Sample Collection for Sequencing

Random sampling of SARS-CoV-2 PCR-positive respiratory samples collected via a convenient sampling design from major testing laboratories covering the majority of Israel has been conducted in Israel as part of the national effort for monitoring SARS-CoV-2 variants, starting in December 2020. Likewise, extracted nucleic acids from SARS-CoV-2 PCR-positive sewage samples collected for environmental surveillance of circulating viruses [17] were used for WGS.

### 2.2. Library Preparation and Sequencing

Viral genomes were extracted from 200 µL respiratory samples with a MagNA PURE 96 (Roche, Germany), according to the manufacturer instructions. Libraries were prepared using a COVID-seq library preparation kit, as per manufacturer’s instructions (Illumina). Library validation and mean fragment size were determined using TapeStation 4200 via DNA HS D1000 kit (Agilent). Libraries were pooled, denatured, and diluted to 10 pM and sequenced on NovaSeq (Illumina, San Diego, CA, USA).

### 2.3. Bioinformatics Analysis

Fastq files were subjected to quality control using FastQC (www.bioinformatics.babraham.ac.uk/projects/fastqc/, accessed on 15 February 2021) and MultiQC [18], and low-quality sequences were filtered using trimmomatic [19]. Sequences were mapped to the SARS-CoV-2 reference genomes (NC_045512.2) using the Burrows–Wheeler aligner (BWA) mem [20]. Resulting BAM files were sorted, indexed, and subjected to quality control using SAMtools suite [21]. Coverage and depth of sequencing was calculated from sorted BAM files using a custom python script. Consensus fasta sequences were assembled for each sample using iVar (https://andersen-lab.github.io/ivar/html/index.html, accessed on 1 June 2021), with positions with <5 nucleotides determined as Ns. Multiple alignment of sample sequences with SARS-CoV-2 reference genome (NC_045512.2) was performed using MAFFT [22].

Mutation calling, translation to amino acid, and identification of P681H variant sequences were carried out in R with a custom code using the Bioconductor package Seqinr [23]. Sequences were further analyzed together with additional sequences identified as belonging to the background lineage B.1.1.50 downloaded from GISAID [2].

Phylogenetic trees were constructed using the Augur pipeline [3]. Sequences were aligned to the SARS-CoV-2 reference genome (NC_045512.2) using MAFFT [22], and a time-resolved phylogenetic tree was constructed with IQ-Tree [24] and TreeTime [25] under the GTR substitution model and visualized with auspice [3]. Lineage nomenclature was attained from Pangolin Lineages [4].

Analysis of sewage samples was conducted using a custom R code and the Bioconductor package Rsamtools [26]. BAM files were imported to R and the frequency of each mutated position along the genome, out of the total number of nucleotides covering that position, was obtained. The frequency of the P681H mutation in the spike protein was recorded for each sample.

### 2.4. Neutralization Assays

VERO-E6 cells at a concentration of 20*10^3^/well were seeded in sterile 96-wells plates with 10% FCS MEM-EAGLE medium, and stored at 37 °C for 24 h. One hundred TCID50 of B.1.1.50 + P681H variant, a strain commonly circulating in Israel lacking a P681H mutation (Israel WT), and B.1.1.7 variant isolates were incubated with inactivated sera from fully vaccinated individuals diluted 1:10 to 1:1280 in 96 well plates for 60 min at 33 °C. Virus–serum mixtures were added to the VERO E6 cells and incubated for five days at 33 °C and stained with Gentain violet staining (1%) to identify the highest serum dilution having no viral cytopathic effect. A dilution equal to 1:10 or above was considered neutralizing. 

## 3. Results

### 3.1. Characterization of the B.1.1.50 + P681H Variant

**Figure 1:**
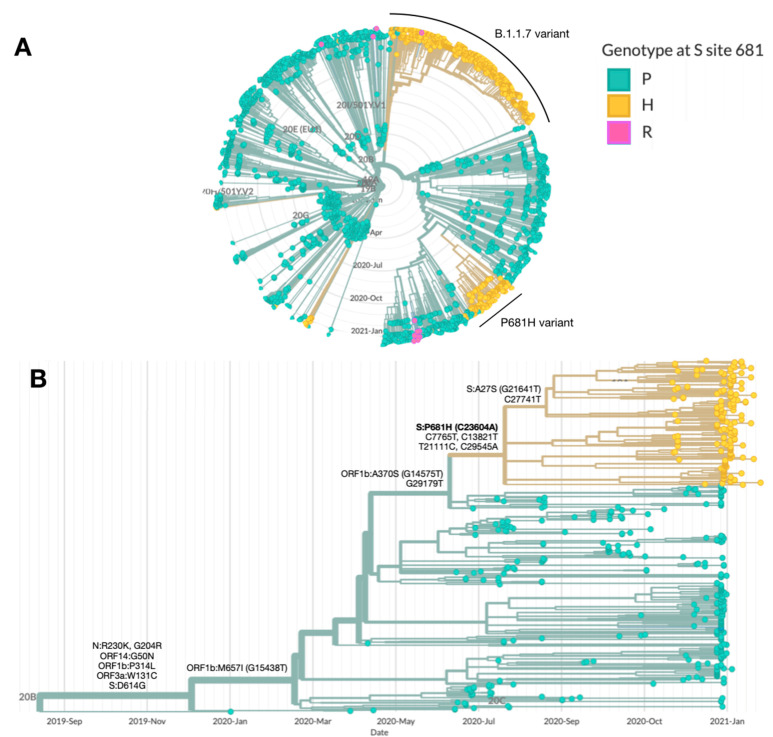
**Characterization of the B.1.1.50 + P681H variant.** Phylogenetic trees highlighting the genotype at site 681 in the S protein, with the wild-type proline (P) in green and the mutations histidine (H) and arginine (R) in yellow and blue, respectively. (**A**) SARS-CoV-2 genomes sequenced in Israel from March 2020 to January 2021 (*n* = 2482). The main clusters harboring the P681H mutation (in yellow) are the B.1.1.7 (20I/501Y.V1) and the B.1.1.50 + P681H variant. (**B**) SARS-CoV-2 genomes from of the B.1.1.50 lineage only (*n* = 489). The B.1.1.50 + P681H cluster is composed of local (Israel) viruses only, with the exception of 2 sequences from isolates identified in the Palestinian authorities, and includes a sub-cluster with an additional S protein non-synonymous mutation, A27S. Additional mutations are listed by each branch. Phylogenetic trees were created with Nextstrain Augur pipeline and visualized with Auspice [3]. Non-Israeli B.1.1.50 lineage sequences were downloaded from GISAID and identified with Pangolin classification [4].

Phylogenetic trees representing randomly chosen samples sequenced in Israel are routinely constructed as part of the national effort for SARS-CoV-2 sequencing and variant surveillance. Two distinct clusters, each with the P681H mutation, were observed; a large cluster, representing the B.1.1.7 variant and a separate smaller cluster emerging from the B.1.1.50 lineage with the P681H mutation (Figure 1A). B.1.1.50 is a locally circulating lineage, in which the majority of the sequences are from Israel (70%), in addition to sequences from the Palestinian authorities (12%) and the UK (12%) [4]. The B.1.1.50 + P681H variant is characterized by the non-synonymous S protein mutation P681H (C23604A) and four additional synonymous mutations: Nsp3:C7765T, Nsp12b:C13821T, Nsp16:T21111C, and C29545A. The B.1.1.50 + P681H variant is unique to Israel, aside from 2 sequences originating in the Palestinian authorities, sharing a border with Israel. A sub-lineage of the variant contains an additional non-synonymous mutation in the S protein at position A27S (G21641T) (Figure 1B). 

Up to January 2021, a total of 181 individuals were detected with the B.1.1.50 + P681H variant. Most of these individuals were randomly sampled from various districts across Israel, with no gender or age bias. The prevalence of the variant in the population decreased with time, from 12.7% out of 212 randomly sequenced sampled in November 2020, to 8.3% in December 2020 (out of 854 random samples), to 3.3% in January (out of 2522 random samples) (Table 1). On the other hand, the B.1.1.7 variant, first identified in Israel in December 2020 and now the dominant lineage in Israel, increased with time, from 0% in November to ~40% in January.

### 3.2. Identification of the P681H Mutation in Sewage

**Figure 2:**
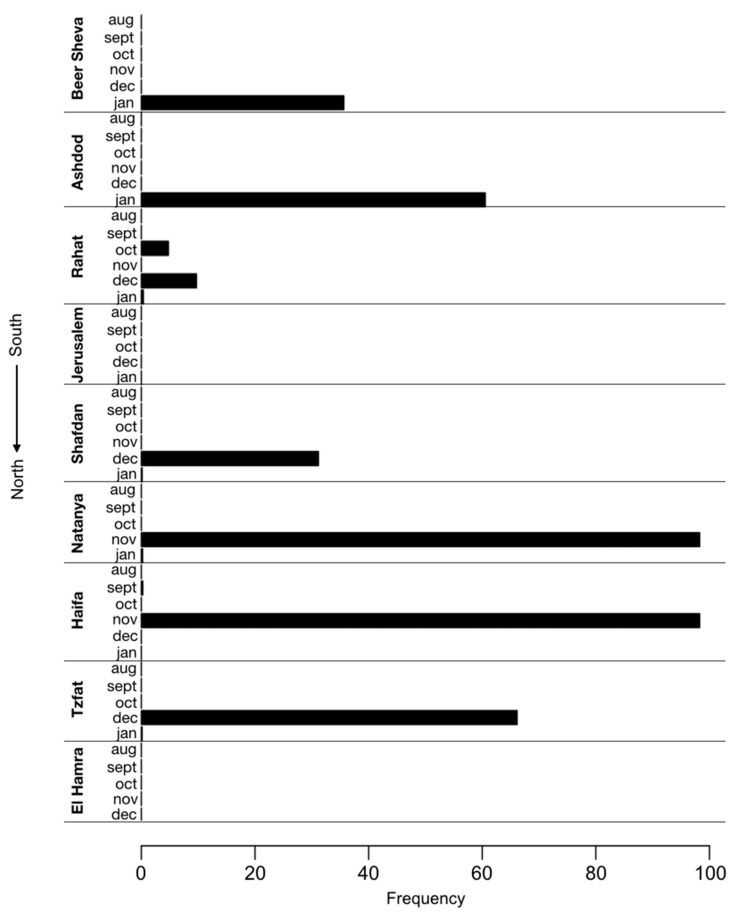
**Identification of the P681H mutation in sewers across Israel.** Frequency of P681H mutation in SARS-CoV-2 genomes sequenced from nine sewage samples of wastewater treatment plants across Israel each month in August 2020–January 2021. The frequency of the P681H mutation in each region was estimated by measuring the fraction of the mutation from the total number of nucleotides mapped at this position (i.e., depth of sequencing).

The P681H mutation was also frequently identified by SARS-CoV-2 whole genome sequencing of samples from waste-water treatment plants in nine locations across Israel, which were collected once a month from August 2020 to January 2021 (Figure 1). As the P681H mutation is also found in the B.1.1.7 variant, its frequency has increased in some locations since late December 2020, following the first introduction of B.1.1.7 into Israel. Notably, the mutation was already observed with a 5% frequency as early as October 2020 in Rahat (city in the South of Israel) and in November, at a frequency of 98% in Natanya and Haifa, located at the center and north of Israel, respectively (Figure 2).

### 3.3. Effective Neutralization of the B.1.1.50 + P681H Variant

**Figure 3:**
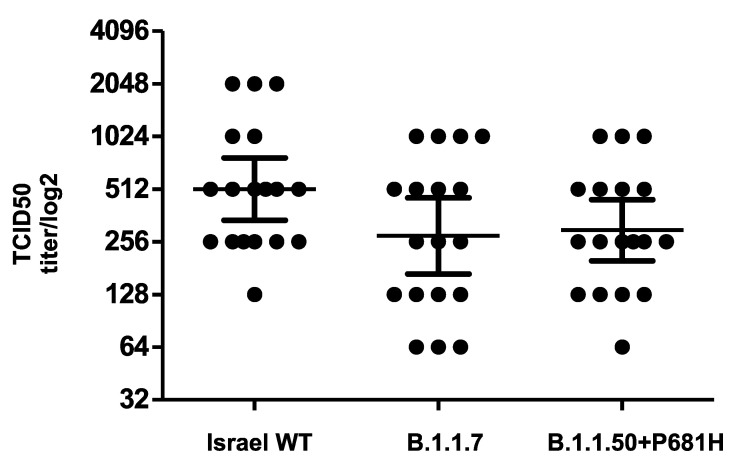
**Neutralization of the B.1.1.50 + P681H variant.** Neutralization assays were carried out with VERO-E6 cells infected with the B.1.1.50 + P681H variant, B.1.1.7 variant and an Israel WT strain from Israel, using sera from fully vaccinated individuals. On day 6, plates were colorized overnight with Gentian violet +4% formaldehyde solution for virus neutralization. Titers were calculated by qualitative measurements of the cytopathic effect for each patient. Bars represent the geometric mean titer (GMT) and 95% confidence intervals.

The neutralization potency of antibodies against the B.1.1.50 + P681H variant was compared to the neutralization of other strains commonly circulating in Israel. VERO-E6 cells were infected with (a) the B.1.1.50 + P681H variant (isolate hCoV-19/Israel/CVL-45176-P681H-ngs/2020), (b) an Israel WT strain (isolate hCoV-19/Israel/CVL-45526-ngs/2020), and (c) the B.1.1.7 variant (isolate hCoV-19/Israel/CVL-46879-ngs/2020). The different strains were pre-incubated with serial dilutions of serum samples obtained from individuals fully vaccinated with two doses of the Pfizer vaccine. The results demonstrate comparable neutralizations of the sera against the B.1.1.50 + P681H variant, the B.1.1.7 variant, and an Israel WT strain (Figure 3). Moreover, negative sera from 10 individuals drawn prior to the pandemic showed no cross-reactivity with any of the virus isolates. 

## 4. Discussion and Conclusions

The P681H mutation was observed as early as March 2020 in samples worldwide and also characterizes the globally spreading B.1.1.7 variant. Herein, we report a novel local variant with the P681H mutation that emerged from the locally circulating B.1.1.50 lineage. This local B.1.1.50 + P681H variant was identified in 181 sequenced samples collected from November 2020 to January 2021 in Israel. Although first detected in clinical samples in November 2020, the P681H mutation had already been identified in Israel in October 2020 via sequencing of sewage samples. 

The 681 site is located in the vicinity of the furin cleavage site in the S protein. It is postulated to enhance transmissibility of the virus by facilitating a conformational change in the S protein following protease activity at the cell membrane [15]. Previous serum neutralization assays for VSV-based SARS-CoV-2 pseudoviruses expressing the S protein with the P681H mutation demonstrated no significant changes in the titer required for neutralization compared to the unmutated S protein [27]. Additionally, no significant changes in the titer were needed for neutralization of the WT SARS-CoV-2 compared to a viral sample from the B.1.1.7 variant (in which P681H is one of its defining mutations), using sera from convalescent patients or individuals vaccinated with the Moderna mRNA-1273 vaccine from Moderna [28]. Similar to these reports, this study demonstrates comparable neutralization of the B.1.1.50 + P681H variant, an Israel WT strain and the B.1.1.7 variant, by sera from vaccinated individuals.

The prevalence of the B.1.1.50 + P681H variant declined with time, from ~12% in November 2020 to 3.2% in January 2021 and 0.9% in February 2021 (out of 2843 randomly sequenced samples, data not shown). The decline of this variant along with additional local lineages, such as B.1.1.50 and B.1.362, is attributed to the increase in prevalence of the B.1.1.7 variant, which was first detected in Israel in December 2020 and is now the dominant variant in Israel, as in many other countries worldwide.

Overall, as the B.1.1.50 + P681H variant was efficiently neutralized by vaccine-derived antibodies, similarly to local unmutated circulating strains, it was not associated with escalated infection or spread, and in light of the P681H mutation being observed in additional SARS-CoV-2 strains worldwide not defined as VOCs, this emerging local variant is currently not defined as a VOC. Nevertheless, it is still monitored by routine next generation sequencing in Israel.

## Figures and Tables

**Table 1:** **Epidemiology of the B.1.1.50 + P681H variant.** Frequency and patient-related information of the B.1.1.50 + P681H variant. Ret. abroad is return from abroad; Jud & Sam is Judea and Samaria.

		November	December	January
gender	Male	10	30	25
	Female	8	24	23
	Unknown	9	17	35
age		39.3 ± 21.3	33.8 ± 25.3	35.8 ± 22
reason	random	27	55	80
	outbreaks		10	
	ret. abroad		6	3
district	Northern	3	7	14
	Haifa	4	2	5
	Central	4	19	32
	Tel Aviv	1	11	10
	Jerusalem	1	9	9
	Jud & Sam	1		5
	Southern	1	4	3
	Unknown	12	19	5
total B.1.1.50 + P681H	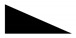	27	71	83
**% of random**		**12.74**	**8.31**	**3.29**
total B.1.1.7	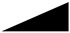	0	64	1008
% of random		0.00	7.49	39.97

## Data Availability

All sequence was deposited to GISAID.
